# 1275. Impact of a bundled antimicrobial stewardship intervention leveraging frontline pharmacist response to rapid blood culture diagnostics at an academic medical center and community hospital

**DOI:** 10.1093/ofid/ofad500.1115

**Published:** 2023-11-27

**Authors:** Kevin Bajer, Christie M Bertram, Erin Weslander, Chao Qi, William J Moore, Radhika S Polisetty, Mike Postelnick, Teresa Zembower, Stephanie Wronkiewicz, Nathaniel J Rhodes, Kendall Kling, Sarah Sutton

**Affiliations:** Northwestern Memorial Hospital, Chicago, Illinois; Northwestern Memorial Hospital/Rosalind Franklin University of Medicine and Science, Chicago, Illinois; Northwestern Memorial Hospital, Chicago, Illinois; Northwestern University Feinberg School of Medicine, Northwestern Memorial Hospital, Chicago, IL; Northwestern Medicine, Chicago, Illinois; Midwestern University College of Pharmacy/ Northwestern Medicine Central DuPage Hospital, Winfield, Illinois; Northwestern Medicine, Chicago, Illinois; Northwestern University, Chicago, Illinois; Northwestern Memorial Hospital, Chicago, Illinois; Midwestern University, Downers Grove, Illinois; Northwestern University, Chicago, Illinois; Northwestern Memorial Hospital, Chicago, Illinois

## Abstract

**Background:**

Rapid Blood Culture Identification (BCID) panels have been shown to improve patient outcomes, including mortality and length of stay, when paired with antimicrobial stewardship (ASP) intervention. We report the results of a quality improvement initiative to expand to 24/7 pharmacist response to BCID results in a large academic medical center and a community hospital within the same health system.

**Methods:**

A pre/post review was performed following a bundled stewardship intervention. Prior to the intervention, BCID results were addressed by the antimicrobial stewardship team Monday through Friday between 8am and 4pm. The bundled ASP intervention consisted of standardized treatment guidance, clinical education on result interpretation, frontline pharmacist evaluation of BCID results during off-peak hours to provide 24/7 response, and electronic health record documentation at each site. Clinical and microbiological data were collected for all patients. The primary outcome was time to optimal therapy (TTOT) for patients with a positive BCID within the first 72 hours of hospital admission. The secondary outcome assessed observed to expected length of stay (O/E LOS) ratios based on Vizient® Clinical Database estimates. The pre-intervention period included patients with a positive BCID between 1/1/2021 and 10/14/2022. The post-intervention period included patients with a positive BCID between 1/3/2023 and 3/31/2023.

**Results:**

Clinical data and outcomes are reported in Table 1. During the first three months following ASP intervention, TTOT decreased at the academic medical center (15.0 to 6.4 hours) but did not change at the community hospital (25.9 to 25.1 hours). The O/E LOS ratio decreased for the academic medical center from 0.92 to 0.78 while the O/E LOS remained similar (0.85 to 0.83) for the community hospital.

**Figure:**
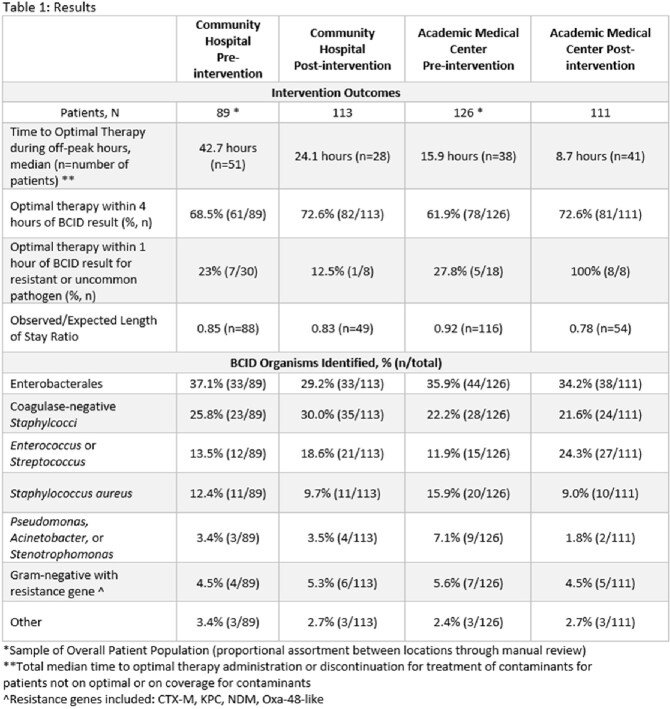

**Conclusion:**

A bundled ASP intervention utilizing frontline, non-ASP pharmacist review and prescriber outreach of BCID results quickly adopted. The intervention demonstrated benefit in the academic medical center, including quicker time to appropriate therapy and reduced length of hospitalization. Additional interventions may be needed for community sites depending on existing resources, staffing models and pharmacist/physician experience.

**Disclosures:**

**Nathaniel J. Rhodes, PharmD MS**, Third Pole Therapeutics: Advisor/Consultant

